# Prognostic factors and surgical approaches in the analysis of primary central nervous system diffuse large B-cell lymphoma: a large population-based cohort study and external validation

**DOI:** 10.3389/fneur.2024.1431614

**Published:** 2024-11-28

**Authors:** Zhibo Pan, Zhaoneng Huang, Zhenqiu Xing, Jianjing Yang, Shengwei Huang, Yu Zhang

**Affiliations:** Department of Neurosurgery, The First Affiliated Hospital of Wenzhou Medical University, Wenzhou, China

**Keywords:** PCNS-DLBCL, prognostic factor, SEER, surgical approaches, nomogram

## Abstract

**Introduction:**

This study aims to investigate prognostic indicators and assess surgical interventions’ impact on Primary central nervous system lymphoma-diffuse large B-cell lymphoma (PCNS-DLBCL) patients.

**Methods:**

A comprehensive examination was performed on a group of 3,962 cases in the Surveillance, Epidemiology, and End Results (SEER) database, as well as 27 cases of PCNS-DLBCL from the First Affiliated Hospital of Wenzhou Medical University. The application of both univariate and multivariate Cox regression analyses facilitated the identification of significant risk factors associated with PCNS-DLBCL. Developing and verifying nomograms, the reliability of the nomogram was evaluated by C-index, ROC curve, calibration curve and decision curve analysis. Finally, by using Kaplan–Meier (KM) curves to assess the survival rates for PCNS-DLBCL patients.

**Results:**

Age, gender, marital status, tumor location, HIV infection status, chemotherapy, and surgical scopes emerged as independent prognostic factors for overall survival (OS) in multivariate Cox regression analysis, whereas gender did not demonstrate significance as a factor for cancer-specific survival (CSS). The C-index, calibration curves, ROC curves, and DCA curves demonstrating strong reliability and practicality. KM analysis revealed significantly improved OS and CSS in patients who underwent surgical resection compared to those who received no surgery/biopsy, especially receiving subtotal resection (STR). In addition, among patients receiving chemotherapy, both STR and gross total resection (GTR) improved survival time compared to chemotherapy alone, particularly with STR. In the non-chemotherapy group, GTR potentially improved CSS, there was no notable disparity in OS between patients who underwent surgery and those who did not or received biopsy.

**Conclusion:**

This study analyzed prognostic factors in PCNS-DLBCL patients, resulting in nomograms predicting 1-, 3-, and 5-year OS and CSS, which showed preferable performance. Combining different resection scopes with chemotherapy improved survival compared to chemotherapy alone, advocating for integrated treatment strategies. Surgery alone is not recommended based on our findings.

## Introduction

Primary central nervous system lymphoma (PCNSL), an infrequent and fatal form of lymphoma confined exclusively to the central nervous system, typically presents with a 5-year survival rate ranging below 30–40% (1), over than 90% of instances are identified as Diffuse Large B-cell Lymphoma (DLBCL) (2, 3). Currently, it is commonly accepted to administer high-dose methotrexate (HD-MTX) during the induction phase is reasonable for newly diagnosed Primary central nervous system lymphoma-diffuse large B-cell lymphoma (PCNS-DLBCL) (4, 5). Nevertheless, a small percentage of patients achieve long-term survival, exhibiting a median progression-free survival of merely 24 months and an overall survival (OS) of approximately 36.9 months (6). Surgical resection has been widely accepted as a crucial component of standard treatment for brain malignant tumors, including gliomas and brain metastases, benefiting to alleviate the mass effect of the tumor, the removal of lesions is considered beneficial for tumor control, potentially providing patients with a certain survival advantage (7). Approximately 50–70% of PCNSL cases are solitary tumors, often located supratentorial. Traditionally, it was believed that surgical treatment did not confer significant survival benefits, with no apparent difference in median survival time between surgical resection and biopsy. As a result, surgery was not considered a standard treatment for PCNSL. However, with advancements in treatment approaches and techniques, this perspective is undergoing a transformation. Certain studies have indicated that surgical resection may prolong the survival period for specific patient populations (4). Hence, this study aims to investigate the clinical features and prognostic determinants of PCNS-DLBCL utilizing information extracted from the Surveillance, Epidemiology, and End Results (SEER) database and the First Affiliated Hospital of Wenzhou Medical University. It will construct a corresponding clinical prognostic prediction model and explore whether different surgical resection scopes, as well as the inclusion of chemotherapy, can bring benefits to the OS and cancer-specific survival (CSS) of PCNS-DLBCL patients.

## Materials and methods

### Patients collection

A retrospective analysis was conducted on pertinent data concerning 3,962 histologically confirmed PCNS-DLBCL patients using the SEER database (released in Nov 2022) from 2000 to 2020. Participants were chosen according to ICD-O-3, using major anatomical site codes (C70.0–C71.9) and histology codes (9,680, Diffuse Large B-cell Lymphoma, NOS, 9684, Malignant Diffuse Large B-cell Lymphoma, Immunoblastic; 9,688, Diffuse Large B-cell Lymphoma with T-cell/Histiocyte Rich Features) for selection.

The data for the external validation set in this study were obtained from the First Affiliated Hospital of Wenzhou Medical University.

### Inclusion criteria

1. Clinically diagnosed with PCNS-DLBCL.
2. Diagnosed when older than 18 years.
3. Defined survival time and follow-up time.

### Exclusion criteria

1. Patients whose diagnosis was confirmed by autopsy or death certificate.
2. Any missing information.

**Figure 1:**
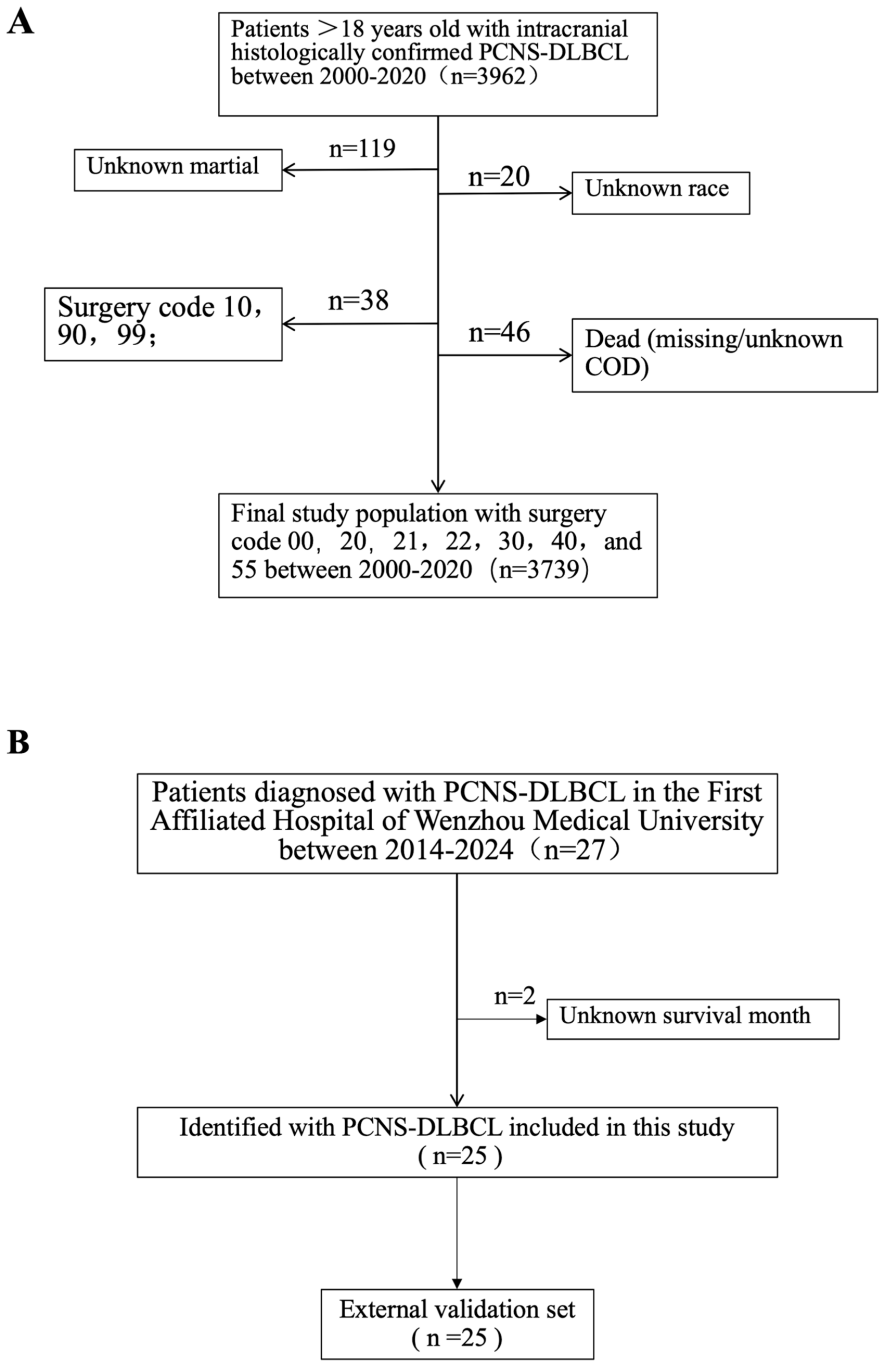
Flowchart illustrating the criteria for selecting 3,739 patients with PCNS-DLBCL from SEER database **(A)**, and 25 patients with PCNS-DLBCL from the First Affiliated Hospital of Wenzhou Medical University **(B)**, were the external validation set.

Following screening, 3,739 patients was selected from the initial PCNS-DLBCL cohort (Figure 1).

### Variable selection and design

**Figure 2:**
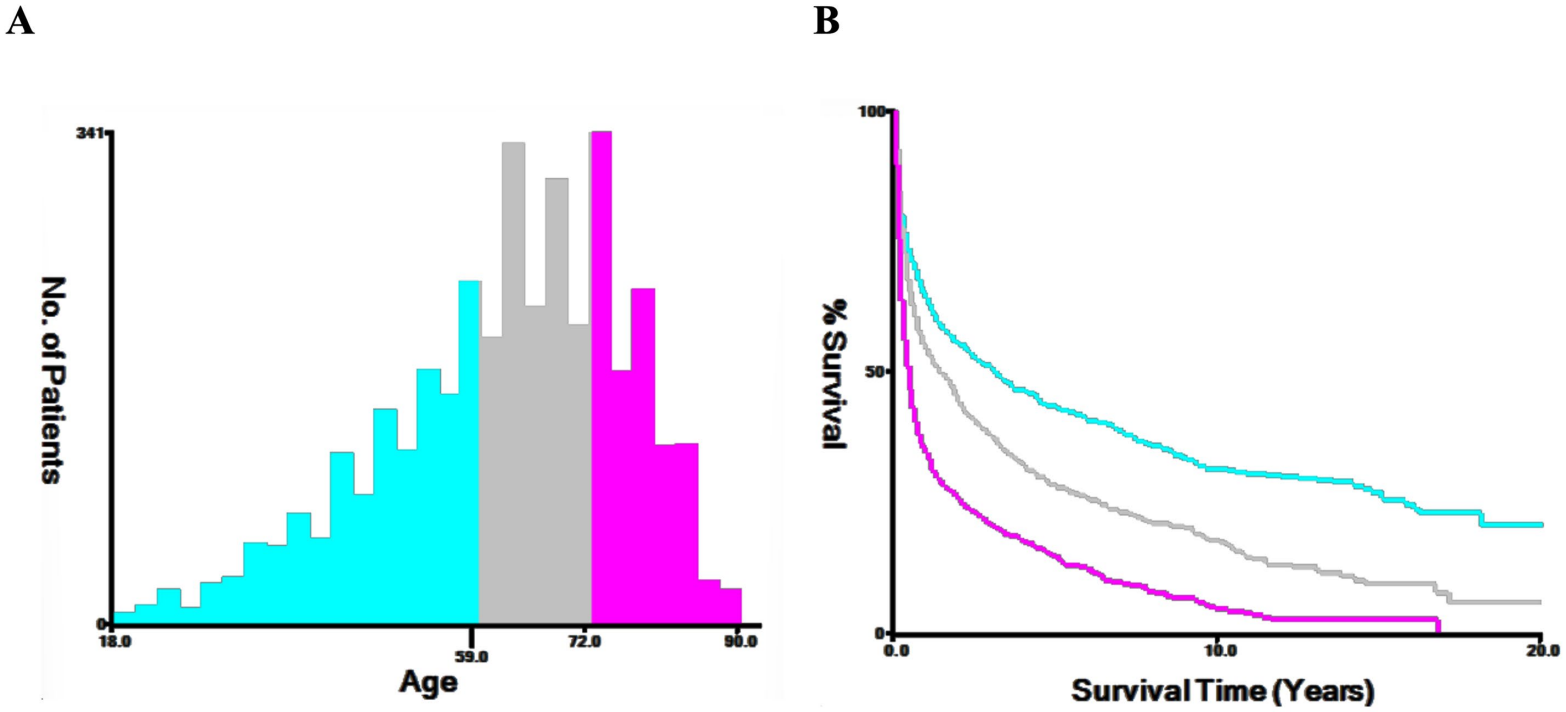
The X-tile procedure’s findings for the optimal age cutoff points. The optimal threshold for age is depicted through a histogram **(A)** and a Kaplan–Meier plot **(B)**, with individuals aged 18–59 in blue, 60–72 in gray, and over 73 in purple. The figure highlights the optimal age thresholds for PCNS-DLBCL at 60 and 73 years.

The patient’s population statistics variables: age, gender, year of diagnosis, marital status, race, lesion location, surgical approach, radiotherapy, chemotherapy status, and HIV infection status. Lesion location was categorized into supratentorial lobes (C71.0-C71.5), cerebellum and brainstem (C71.6, C71.7), overlapping (C71.8), and Brain, NOS (C71.9). The surgical approach was categorized as: no surgery/biopsy (codes 00 and 20), subtotal resection (STR) (codes 21, 22, 40, and 90), and gross total resection (GTR) (codes 30 and 55). Continuous variables were ascertained employing the X-tile technique to pinpoint the optimal cut-off point, while age was stratified into three cohorts: 18–59 years, 60–72 years, and ≥ 73 years (Figure 2).

### Statistical analysis

Conducting *χ*^2^ tests, along with univariate and multivariate Cox regression analyses, this study assessed clinical variables in both training and validation sets. The objective was to pinpoint significant risk factors and independent prognostic variables linked to OS and CSS within the training set. Utilizing the outcomes derived from the multivariate Cox regression, nomograms were developed, and the effectiveness and clinical utility of the nomogram were assessed using the Concordance Index (C-index), Receiver Operating Characteristic (ROC) curves, calibration curves, and decision calibration curves (DCA).

Kaplan–Meier curves were employed to analyze the survival characteristics of OS and CSS under different treatment regimens. Data analysis for this study was conducted using Excel 2024 and R software (version 4.3.2). The statistical significance was regarded when the significance level of *p* < 0.05 was met. Datasets of training set and validation set were obtained from the SEER*Stat (version 8.4.2), released in Nov 2022. The external validation set was obtained from the First Affiliated Hospital of Wenzhou Medical University.

## Results

### Patient characteristics

This investigation involved 3,739 patients identified with PCNS-DLBCL from the SEER database, with 2,617 patients in the training group and 1,122 in the validation group, with 25 patients in the external validation group. The specific attributes of the included participants are delineated in Table 1.

**Table 1:** Demographic and clinicopathologic profiles of patients in primary central nervous system diffuse large B-cell lymphoma.

	[ALL]	Training set	Validation set	*p* value	External validation
	*N* = 3,739	*N* = 2,617	*N* = 1,122		*N* = 25
Age				0.306	
18–59 years	1,331 (35.6%)	911 (34.8%)	420 (37.4%)		8 (32%)
60–72 years	1,349 (36.1%)	957 (36.6%)	392 (34.9%)		9 (36%)
>72 years	1,059 (28.3%)	749 (28.6%)	310 (27.6%)		8 (32%)
Sex				0.270	
Female	1,816 (48.6%)	1,287 (49.2%)	529 (47.1%)		12 (48%)
Male	1,923 (51.4%)	1,330 (50.8%)	593 (52.9%)		13 (52%)
Year of diagnosis				0.792	
2000–2010	1,659 (44.4%)	1,157 (44.2%)	502 (44.7%)		0 (0%)
2011–2020	2,080 (55.6%)	1,460 (55.8%)	620 (55.3%)		25 (100%)
Marital status				0.340	
Single^a^	1,491 (39.9%)	1,030 (39.4%)	461 (41.1%)		0 (0%)
Married	2,248 (60.1%)	1,587 (60.6%)	661 (58.9%)		25 (100%)
Race				0.694	
White	2,947 (78.8%)	2,066 (78.9%)	881 (78.5%)		0 (0%)
Black	231 (6.18%)	156 (5.96%)	75 (6.68%)		0 (0%)
Others^b^	561 (15.0%)	395 (15.1%)	166 (14.8%)		25 (100%)
Location				0.756	
Supratentorial	2,003 (53.6%)	1,410 (53.9%)	593 (52.9%)		11 (44%)
Infratentorial	281 (7.52%)	201 (7.68%)	80 (7.13%)		8 (32%)
Overlapping	381 (10.2%)	267 (10.2%)	114 (10.2%)		6 (24%)
Brain, NOS	1,074 (28.7%)	739 (28.2%)	335 (29.9%)		0 (0%)
Surgery				0.454	
No or biopsy	2,846 (76.1%)	1,977 (75.5%)	869 (77.5%)		9 (36%)
STR	267 (7.14%)	192 (7.34%)	75 (6.68%)		4 (16%)
GTR	626 (16.7%)	448 (17.1%)	178 (15.9%)		11 (44%)
Radiation				0.855	
NO/Unknown	2,672 (71.5%)	1,873 (71.6%)	799 (71.2%)		19 (76%)
YES	1,067 (28.5%)	744 (28.4%)	323 (28.8%)		6 (24%)
Chemotherapy				1.000	
NO/Unknown	1,088 (29.1%)	762 (29.1%)	326 (29.1%)		2 (8%)
YES	2,651 (70.9%)	1,855 (70.9%)	796 (70.9%)		23 (92%)
HIV infection				1.000	
NO	3,536 (94.6%)	2,475 (94.6%)	1,061 (94.6%)		25 (100%)
YES	203 (5.43%)	142 (5.43%)	61 (5.44%)		0 (0%)
Median OS months	13	13	12		17
Median CSS months	17	19	14		12
No. of death (%)	2,635 (70.5%)	1,602 (61.2%)	196 (70.9%)		9 (36%)
5-year OS rate	29.8%	29.9%	29.6%		55.6%
5-year CSS rate	34.5%	34.7%	34.1%		62.6%

a Included Single (never married)/Widowed/Divorced/Separated.

b Included American Indian/AK Native, Asian/Pacific Islander.

Among these patients from the SEER database, 1,331 (35.6%) were aged between 18 and 59, 1,349 (36.1%) were aged between 60 and 72, and 1,059 (28.3%) were 73 years or older. A total of 1,923 (51.4%) male patients were identified, and 1,816 (48.6%) as female, the male-to-female ratio was calculated at 1:0.94. Patients diagnosed before 2010 numbered 1,659 (44.7%), slightly fewer than those diagnosed after 2010, which amounted to 2080 (55.6%). 2,248 (60.1%) patients were married, and 1,491 (39.9%) were singled. Supratentorial tumors accounted for more than half of the cases (53.6%), with Brain, NOS being the next most common location (28.7%). 2,651 (70.9%) patients, received chemotherapy, while only 1,067 (28.5%) received radiotherapy, similarly, in the external validation set, 23 (92%) received chemotherapy. HIV-positive patients accounted for only 203 cases (5.43%). In terms of surgery, a total of 2,846 (76.1%) patients did not undergo surgery or received only a biopsy, 267 (7.14%) underwent STR, and 626 (16.7%) underwent GTR, while 11 (44%) received GTR in external validation set. The clinical features of patients between the training and validation sets exhibited comparability.

### Analysis of influencing factors of OS

The median OS among the 3,739 patients was 13 months. Among them, a total of 2,635 patients died, including 2,302 cancer-specific deaths and 333 deaths from other causes. Patients exhibited varying OS rates at 1, 3, and 5 years, with survival rates of 51.5, 37.4, and 29.8%, respectively.

**Figure 3:**
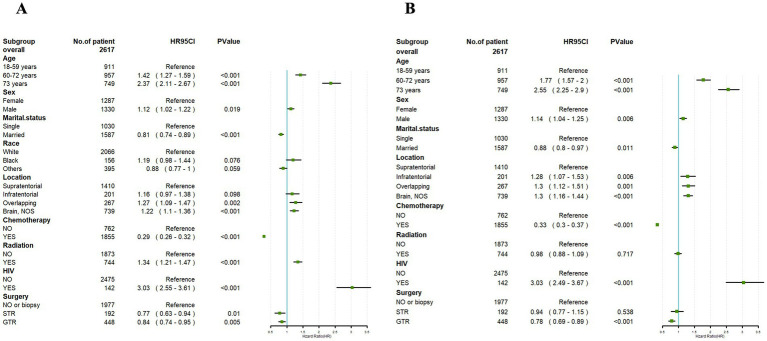
Univariate Cox **(A)** and multivariate Cox **(B)** analyses were conducted to assess the association between clinical factors and overall survival. An invalid line is characterized by a straight line with a coordinate of 1 perpendicular to the *x*-axis. Lines parallel to the *x*-axis signify the 95% confidence interval for each variable. When a line intersects with the invalid line, it suggests a lack of statistical significance (*p* > 0.05).

Univariate Cox regression analysis performed on the training set demonstrated significant correlations between OS and factors such as age, gender, marital status, tumor location, radiotherapy, chemotherapy, HIV infection status, and surgical approach (*p* < 0.05). However, race exhibited no significant association with the OS of PCNS-DLBCL (*p* > 0.05) (Figure 3A).

Building upon the outcomes of the univariate analysis, a multivariate Cox regression analysis was subsequently conducted, followed by the construction of a forest plot. The results revealed that age, gender, marital status, tumor location, chemotherapy, HIV infection status, and surgical approach were identified as independent prognostic factors for OS (*p* < 0.05). Male and HIV-positive were both link to a prognosis. In addition, the age at diagnosis younger than 60 years, married, tumor location in the supratentorial, undergoing chemotherapy, and receiving GTR were correlated with a better prognosis.

However, radiotherapy had no significant impact in the prognosis of PCNS-DLBCL (Figure 3B).

### Analysis of influencing factors of CSS

Among the 2,617 patients in the training set, 1,602 died from PCNS-DLBCL. The CSS of patients at 1, 3, and 5-years were 54.6, 41.4 and 34.5%, respectively.

**Figure 4:**
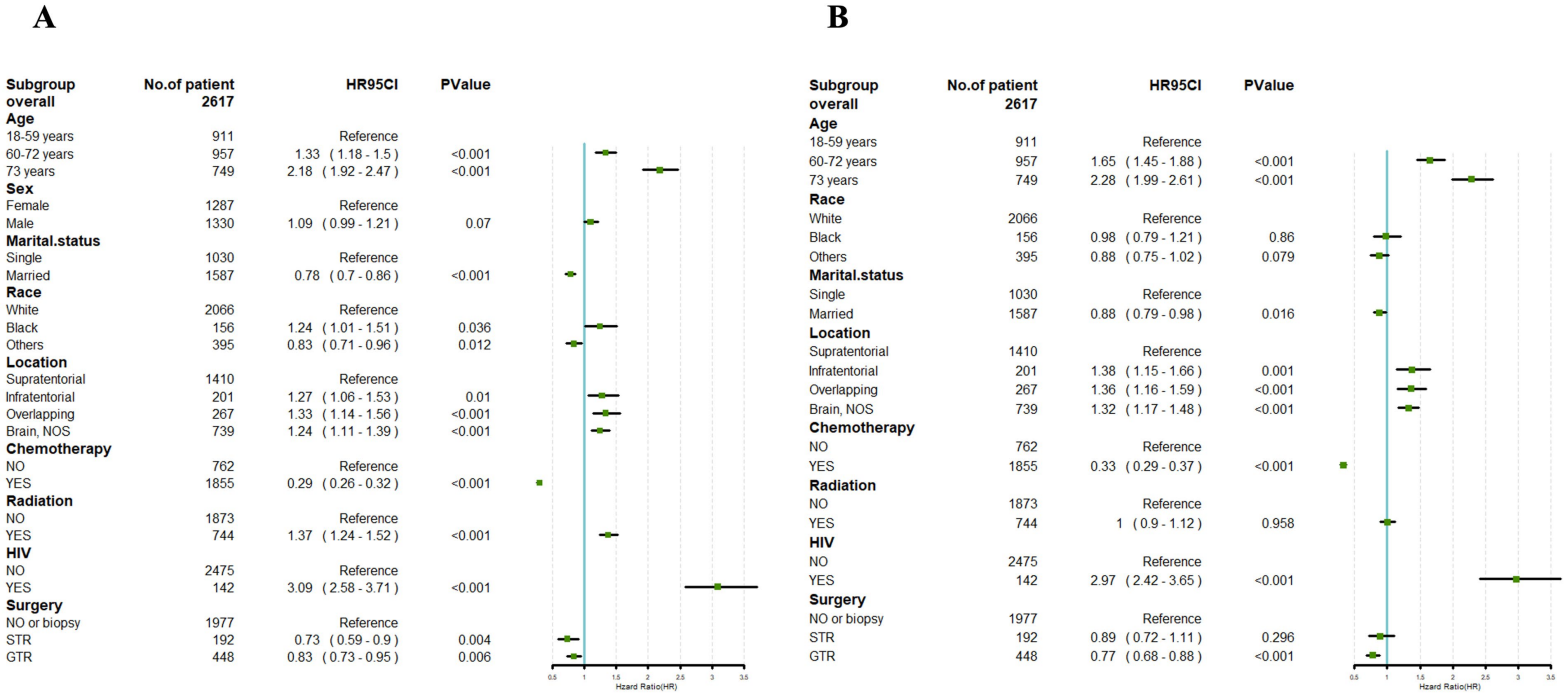
Univariate Cox **(A)** and multivariate Cox **(B)** analyses were conducted to assess the association between clinical factors and cancer-specific survival. An invalid line is characterized by a straight line with a coordinate of 1 perpendicular to the *x*-axis. Lines parallel to the *x*-axis signify the 95% confidence interval for each variable. When a line intersects with the invalid line, it suggests a lack of statistical significance (*p* > 0.05).

Univariate Cox regression analysis performed on the training cohort showed notable correlations between CSS and factors such as age, marital status, tumor location, radiotherapy, chemotherapy, HIV infection status, and surgical approach (*p* < 0.05). However, gender exhibited no significant association with the CSS of PCNS-DLBCL (*p* > 0.05) (Figure 4A).

Building upon the outcomes of the univariate analysis, a multivariate Cox regression analysis was subsequently conducted, followed by the construction of a forest plot. The results revealed that age, marital status, tumor location, chemotherapy, HIV infection status, and surgical approach were identified as independent prognostic factors for CSS (*p* < 0.05). HIV-positive was link to a prognosis. In addition, the age at diagnosis younger than 60 years, married, tumor location in the supratentorial, undergoing chemotherapy, and receiving GTR were correlated with a better prognosis.

However, race and with or without radiotherapy had no significant impact in the prognosis of PCNS-DLBCL (Figure 4B).

### Nomogram construction

**Figure 5:**
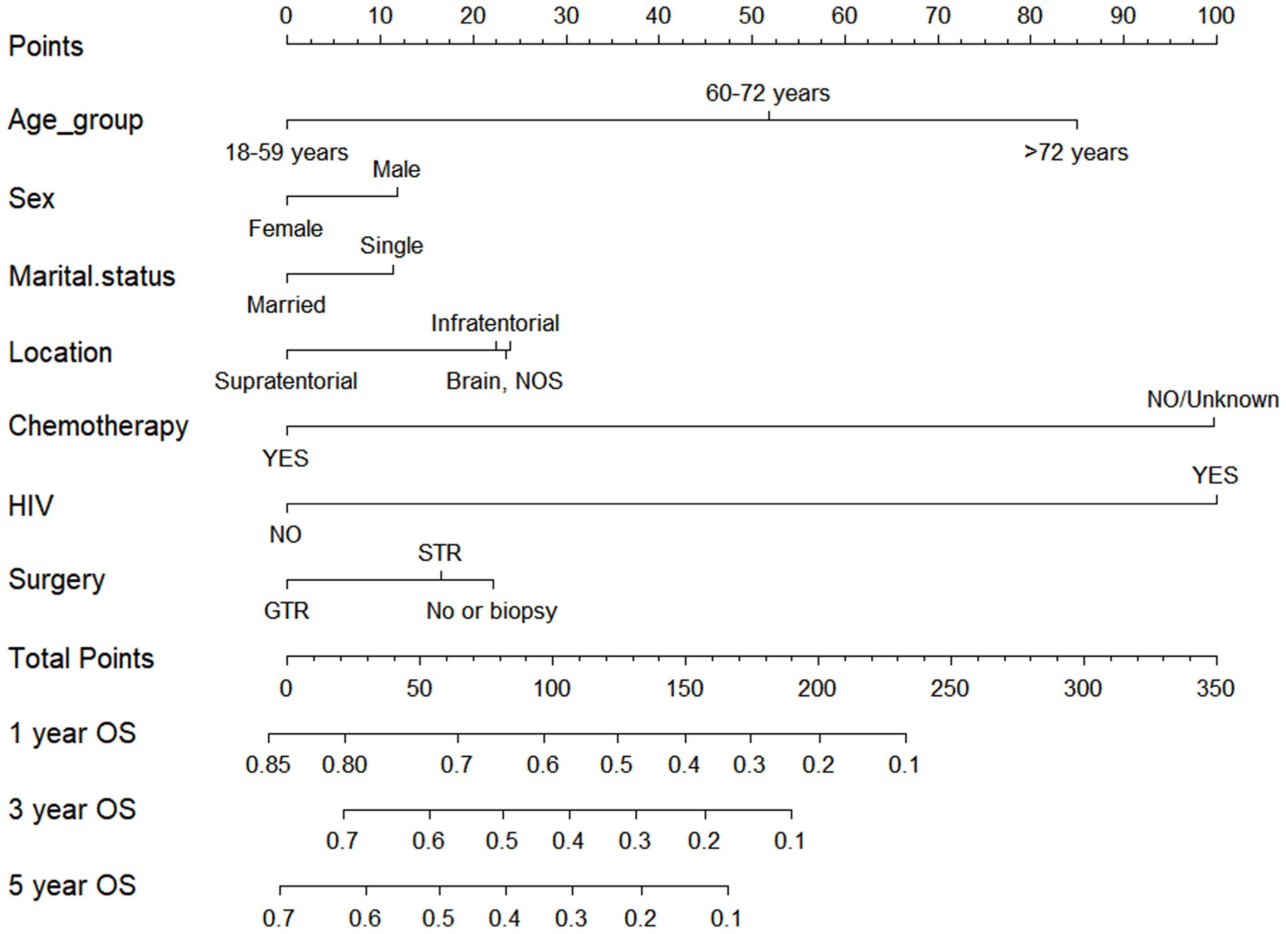
Nomogram for forecasting overall survival (OS) in primary central nervous system- Diffuse large B-cell lymphoma.

According to outcomes of the multivariate Cox regression analysis, significant independent prognostic factors impacting OS were amalgamated to formulate a nomogram. The included prognostic factors included: age, gender, marital status, tumor location, chemotherapy, HIV infection status, and surgical approach. The prediction predictions for OS at 1, 3, and 5-year OS are depicted in Figure 5. The nomogram revealed that patients aged 18–59 years, females, being married, tumors located in supratentorial, with chemotherapy, HIV negative infection, and receiving GTR had lower corresponding scores, indicating higher 1, 3, and 5-year OS.

**Figure 6:**
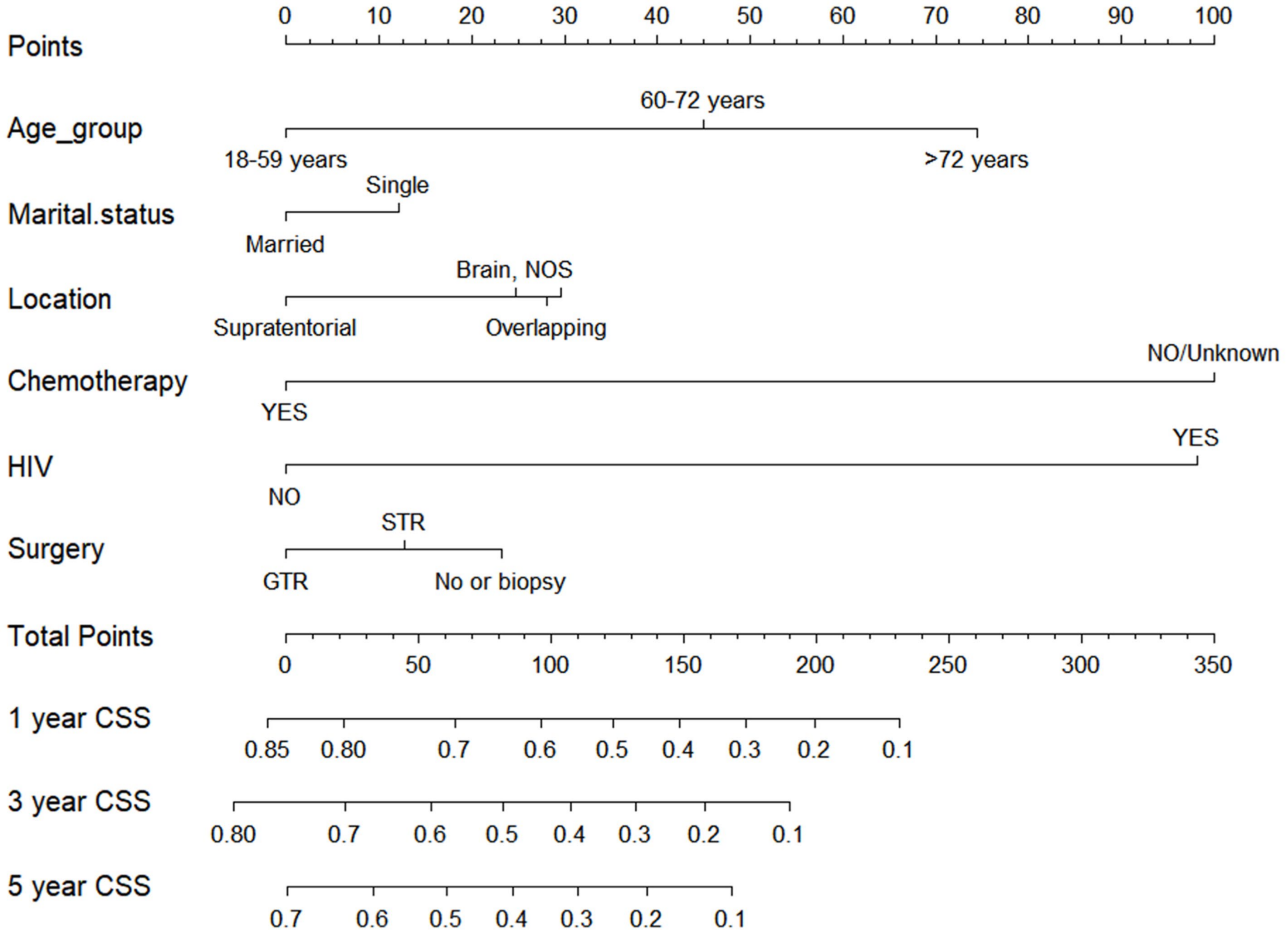
Nomogram for forecasting cancer-specific survival (CSS) in primary central nervous system- Diffuse large B-cell lymphoma (PCNS-DLBCL).

Drawing from the findings of the multivariate analysis, we integrated independent prognostic factors impacting CSS to devise a nomogram, such as age, marital status, tumor location, chemotherapy, HIV infection status, and surgical approach. The prediction predictions for CSS at 1, 3, and 5-year are depicted in Figure 6. The nomogram revealed that patients aged 18–59 years, being married, tumors located in supratentorial, with chemotherapy, HIV negative infection, and receiving GTR had lower corresponding scores, indicating higher 1, 3, and 5-year OS.

### Verification of the nomogram

The validation outcomes revealed a C-index of 0.716 (95% CI: 0.704–0.739) for predicting OS in the training set, 0.704 (95% CI: 0.685–0.724) in the validation set, 0.607 (95% CI: 0.510–0.704) in the external validation set. Additionally, the C-index for forecasting CSS was 0.715 (95% CI: 0.702–0.728) in the training set, 0.696 (95% CI: 0.675–0.717) in the validation set, 0.610 (95% CI: 0.402, 0.818) in the external validation set. These findings suggest that the nomogram exhibits favorable prognostic value for both OS and CSS (Table 2).

**Table 2:** The C-index of nomogram.

Nomogram	Training set (95%CI)	Internal validation set (95% CI)	External validation set (95% CI)
Nomogram on OS	0.716 (0.704, 0.739)	0.704 (0.685, 0.724)	0.607 (0.510, 0.704)
Nomogram on CSS	0.715 (0.702, 0.728)	0.696 (0.675, 0.717)	0.610 (0.402, 0.818)

**Figure 7:**
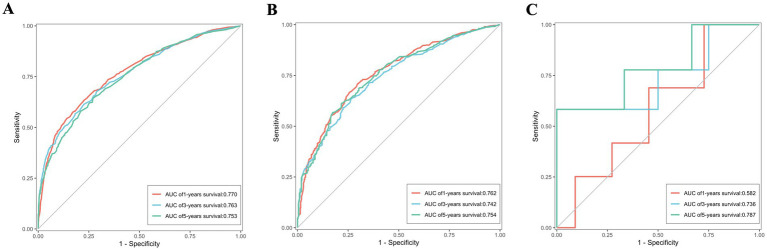
The ROC depicting the nomogram’s predictive accuracy for overall survival as follows: **(A)** The AUC of 1, 3, and 5-year in the training set. **(B)** The AUC of 1, 3, and 5-year in the validation set. **(C)** The AUC of 1, 3, and 5-year in the external validation set.

**Figure 8:**
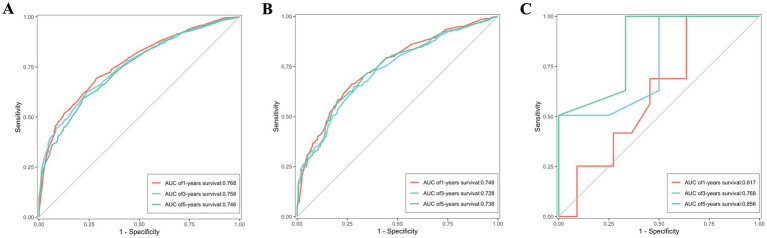
The ROC depicting the nomogram’s predictive accuracy for cancer-specific survival as follows: **(A)** The AUC of 1, 3, and 5-year in the training set. **(B)** The AUC of 1, 3, and 5-year in the validation set. **(C)** The AUC of 1, 3, and 5-year in the external validation set.

ROC were utilized to assess the nomogram’s performance comprehensively. In the training set, the AUC values for predicting 1, 3, and 5-year OS stood at 0.770, 0.763, and 0.753, respectively. Similarly, were 0.762, 0.742, and 0.754 in the validation set, and were 0.582, 0.736 and 0.787 in the external validation set, as depicted in Figures 7A–C. Furthermore, the AUC values for predicting 1, 3, and 5-year CSS in the training set were 0.768, 0.758, and 0.746, respectively, were 0.748, 0.728 and 0.738 in the validation set, and were 0.617, 0.768 and 0.858 in the external validation set, illustrated in Figures 8A–C. These results shown the effectiveness and accuracy of the predictive model.

**Figure 9:**
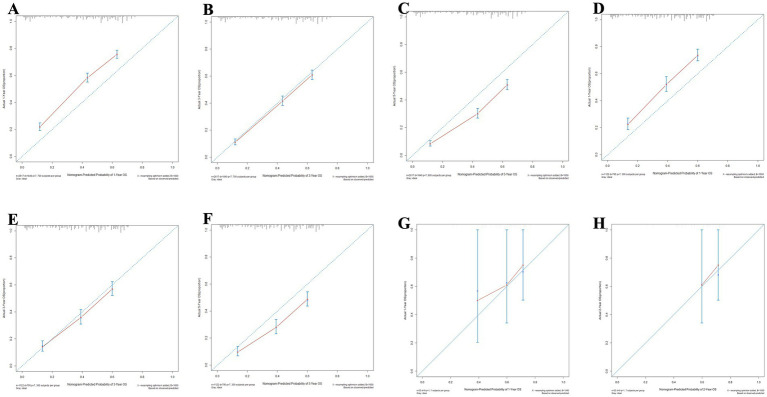
The calibration curves for the nomogram forecasting overall survival (OS) are detailed in panels **(A–C)** for 1, 3, and 5-year OS predictions in the training set, **(D–F)** for the same time points in the validation set, **(G,H)** for 1 and 2-year OS predictions in the external validation.

**Figure 10:**
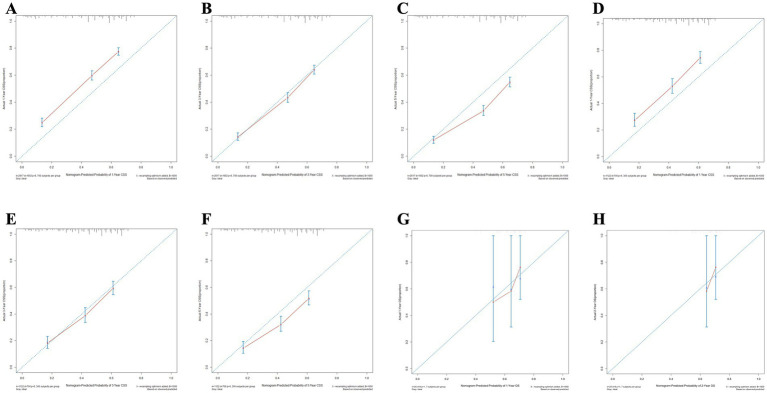
The calibration curves for the nomogram forecasting cancer-specific survival (CSS) are detailed in panels **(A–C)** for 1, 3, and 5-year OS predictions in the training set, **(D–F)** for the same time points in the validation set, **(G,H)** for 1 and 2-year OS predictions in the external validation.

Meanwhile, the calibration curves for forecasting 1, 3, and 5-year OS and CSS are illustrated in Figures 9, 10, respectively. The calibration curves for the training set, validation set, and external validation set exhibit close to the ideal 45° dashed line, indicative of strong alignment between predicted and actual values.

**Figure 11:**
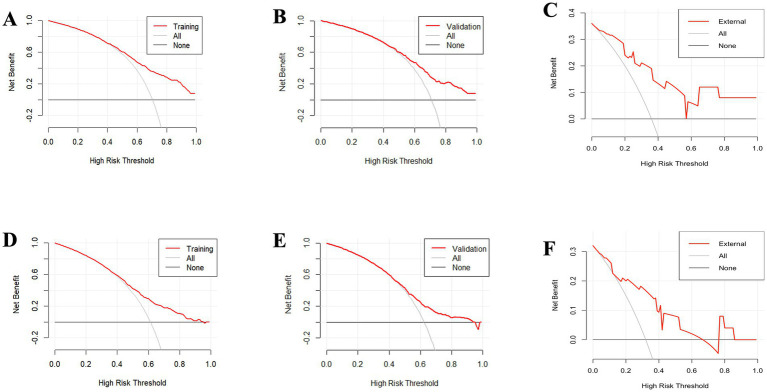
The decision curve analysis (DCA) for forecasting the overall survival (OS) of patients illustrates the model’s effectiveness in both the training set **(A)**, the validation set **(B)**, the external validation set **(C)**. Additionally, the analysis extends to predicting cancer-specific survival (CSS), demonstrating the model’s predictive accuracy in the training set **(D)**, the validation set **(E)**, and the external validation set **(F)**.

Furthermore, the DCA for forecasting the OS and CSS of PCNS-DLBCL was in Figure 11. When the probability thresholds for OS are within the range of 0.4 to 0.9, the net benefit derived significantly surpasses the outcomes of either opting for “no intervention” or “full intervention.” Similarly, for CSS predictions, when probability thresholds range from 0.45 to 0.95, the observed net benefit markedly exceeds the outcomes associated with both the “no intervention” or “full intervention.” This indicates that the nomogram demonstrates substantial clinical utility in forecasting OS and CSS.

### Survival analysis of surgery

**Figure 12:**
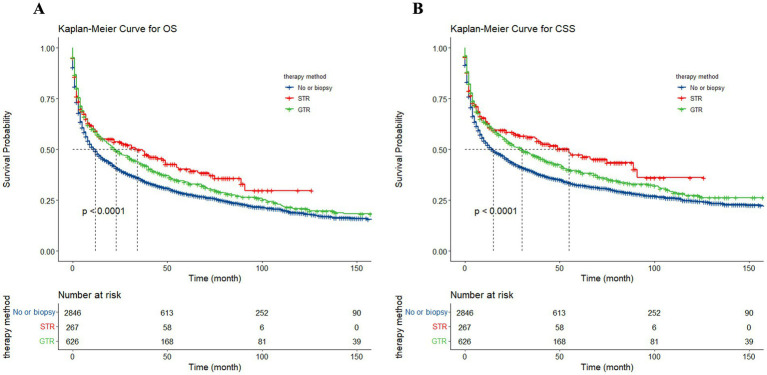
The Kaplan–Meier illustrating the impact of surgical approach on overall survival (OS) **(A)** and cancer-specific survival (CSS) **(B)** for all patients.

**Figure 13:**
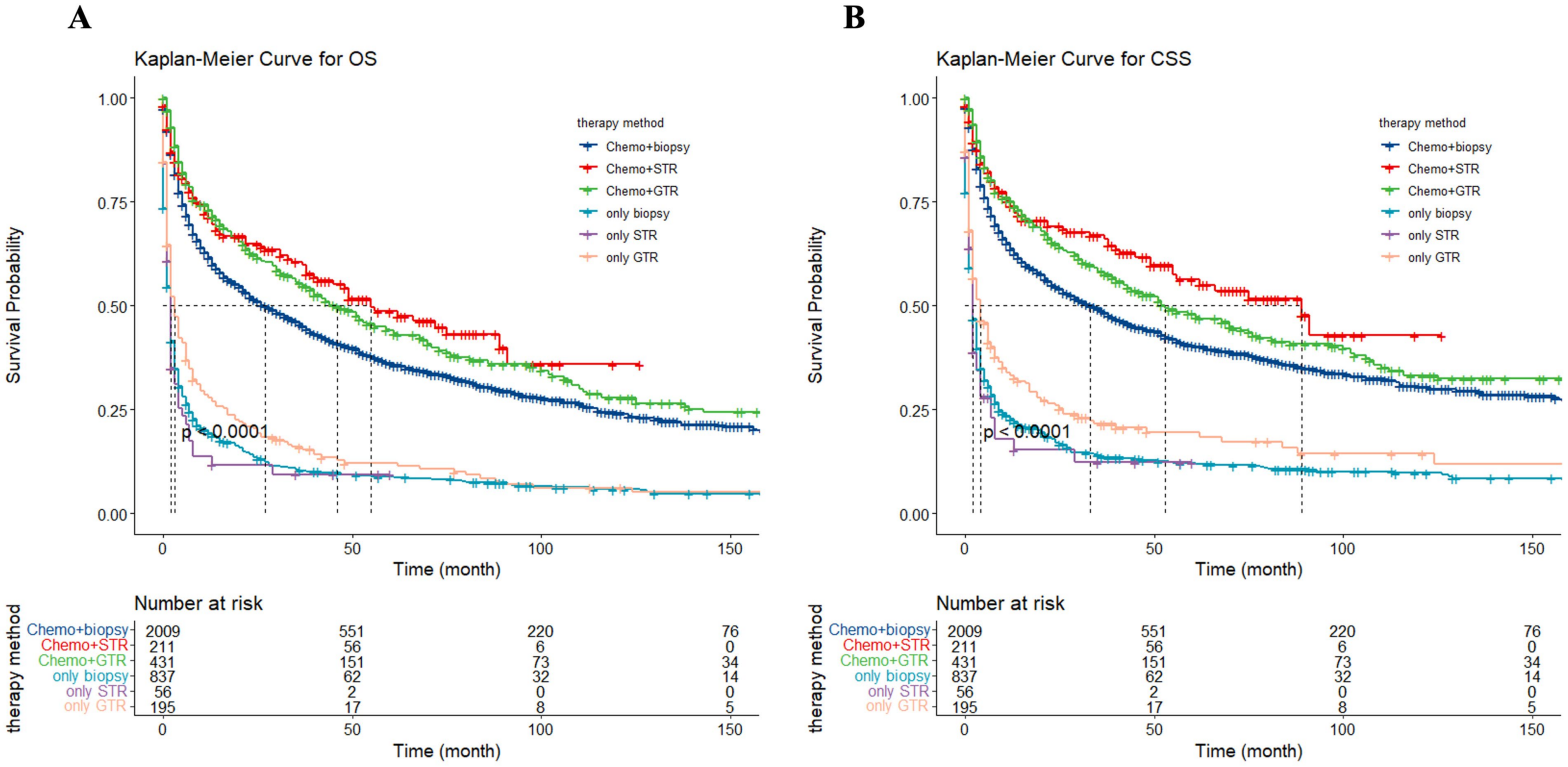
The Kaplan–Meier depicting the influence of treatment strategy on overall survival (OS) **(A)** and cancer-specific survival (CSS) **(B)** for all patients.

To assess the influence of surgical approach and treatment strategy on patient outcomes, the study categorized the 3,739 patients into three distinct cohorts: no surgery/biopsy group (blue), STR group (red), and GTR group (green) (Figure 12). The Kaplan–Meier curves show that patients receive surgery had significantly preferable outcomes compared to those in the no surgery/biopsy. Within the surgery group, individuals undergoing STR had a more favorable prognosis than those undergoing GTR (*p* < 0.0001). The median OS for the STR group (red) was 34 months (95% CI: 15 to 49 months), for the GTR group (green) it was 23 months (95% CI: 18 to 30 months), and for the no surgery/biopsy group (blue) it was 12 months (95% CI: 10 to 13 months) (*p* < 0.0001). Similarly, regarding CSS, the median CSS for the STR group was 55 months (95% CI: 33 to 91 months), for the GTR group it was 30 months (95% CI: 23 to 40 months), and for the no surgery/biopsy group it was 15 months (95% CI: 13 to 18 months) (*p* < 0.0001). We further divided all patients into 6 groups: chemotherapy plus non-surgical/biopsy group (deep blue); chemotherapy plus STR group (red); chemotherapy plus GTR group (green); non-chemotherapy plus non-surgical/biopsy group (light blue); non-chemotherapy plus STR group (purple); non-chemotherapy plus GTR group (pink). Patients who received chemotherapy exhibited significantly better results compared to those who did not (deep blue vs. light blue, red vs. purple, green vs. pink). Among patients receiving chemotherapy, those receiving chemotherapy plus surgery still had better OS and CSS compared to patients receiving chemotherapy alone. Furthermore, the chemotherapy plus STR showed superiority over the chemotherapy plus GTR, while the effectiveness of chemotherapy alone is inferior to the latter. The median OS was 55 months (red); 46 months (green); 27 months (deep blue) (*p* < 0.0001). The median CSS was 89 months (red); 53 months (green); 33 months (deep blue) (*p* < 0.0001). However, among the three groups of patients not receiving chemotherapy, we did not observe significant differences in OS, with median OS of 2 months (light blue); 2 months (purple); 3 months (pink) (*p* = 0.024). However, in terms of CSS, patients in the non-surgical plus GTR group had slightly better prognosis than the other two groups, with median CSS of 4 months (pink); 2 months (light blue); 2 months (purple) (*p* = 0.0064). The reason may be that GTR can alleviate the pressure of the tumor on the local nerves. However, in the long term, patients undergoing GTR exhibited improved OS and CSS compared to the other two groups, but still significantly worse than the group receiving chemotherapy (Figure 13).

## Discussion

Being an uncommon malignancy of the central nervous system, research on PCNS-DLBCL primarily relies on retrospective analysis with small series. The role of surgical treatment remains controversial, and the effectiveness of surgery combined with chemotherapy is still uncertain (5). Thus, we conducted an extensive study based on the SEER database, utilizing data spanning the past 20 years, we analyzed characteristics and treatment strategies of PCNS-DLBCL patients aged 18 and above. Nomograms were developed to predict patient prognosis. Subsequently, both the C-index, ROC, and calibration curves indicated the model’s reliability, while DCA demonstrated its clinical utility. Additionally, the respective 1, 3, and 5-year OS rates in this study were 51.5, 37.4, and 29.8%, while the corresponding CSS rates were 54.6, 41.4, and 34.5%. Kasenda et al. reported a 5-year OS ranging from 15 to 30%, which aligns with our research findings (8, 9). Therefore, the nomogram constructed in this study, incorporating multiple independent risk factors, holds potential clinical significance in enhancing the survival and prognosis of PCNS-DLBCL patients.

In this study, age was identified as a significant independent risk factor, with patient OS and CSS decreasing annually with age, consistent with previous research. We found that patients over 73 years old had a particularly poor prognosis. Firstly, elderly patients have a higher probability of suffering from other primary refractory diseases. Secondly, immune system dysregulation results in a gradual decline in the production of naïve T cells, making them more susceptible to iatrogenic toxicity (10–12). All of this suggests that early diagnosis is crucial for PCNS-DLBCL patients who was suspected to facilitate timely treatment. Furthermore, we also found that unmarried individuals (including Single, divorced, separated, widowed, unmarried or domestic partner) had a poorer prognosis compared to married individuals. This might be associated with the lack of psychological support from family members and differences in social environment among this group of patients (9, 13). It is important to provide more psychological support and comfort for unmarried patients in clinical treatment.

Our findings suggest that tumor location serves as an independent prognostic factor for PCNS-DLBCL, with tumors located deeper having a poorer prognosis. A previous study suggested that through gene expression analysis of patients receiving HD-MTX chemotherapy, signal transduction-related genes such as receptor activity, molecular transduction, and antioxidant activity were upregulated in PCNSL patients with superficial tumors compared to those with deeper tumors, while catalytic activity-related genes were downregulated. This suggests that PCNSL located in superficial brain structures may respond better to HD-MTX alone compared to deeper structures (14). Another retrospective study similarly demonstrated that among patients younger than 70 years old with isolated superficial lesion, those receiving surgical resection experienced significantly prolonged survival compared to those receiving biopsy (15).

Historically, AIDS-related primary central nervous system lymphoma (AR-PCNSL) has been widely viewed as a terminal consequence of HIV infection, with a median survival period rarely exceeding 3 months (16). In this study, the risk of mortality among HIV-positive patients was 3.03 times higher than that among HIV-negative patients (*p* < 0.0001). The reasons could be the more severe immunosuppression, more aggressive lymphoma, or poorer physical condition in patients (17). Currently, it is recommended to combine antiretroviral therapy (cART) with HD-MTX for AR-PCNSL patients. However, the pharmacokinetic parameters of chemotherapy drugs may be interfered with due to interactions with cART. Additionally, combined cART and chemotherapy treatment may lead to a significant decrease in the peripheral blood CD4 and CD8 cell counts. Overall, the prognosis for AR-PCNSL patients is not optimistic (18).

The current consensus is that newly diagnosed PCNSL patients should receive combination therapy based on HD-MTX, and treatment regimens containing rituximab can be administered during the induction therapy (19). In this study, 70.9% of patients received chemotherapy, and it was observed that compared to those who did not receive chemotherapy, patients exhibited significant enhancements in both OS and CSS. In recent years, with the introduction of HD-MTX, although the survival period of PCNS-DLBCL patients has been significantly prolonged, only 50% of patients achieve long-term remission, and so far, there is still no consensus regarding the optimal dosage and regimen for chemotherapy (20, 21). Therefore, there have been numerous prospective studies currently, aimed at exploring the optimal treatment regimens, aiming to alleviate patients’ neurotoxic reactions to improve their prognosis (22–24).

PCNSL exhibits high sensitivity to radiotherapy (6). While whole-brain radiotherapy (WBRT) can effectively reduce lesion size and alleviate symptoms, its radiation toxicity and high recurrence rate contribute to a poor prognosis for patients treated with WBRT alone, with the majority of patients experiencing 5-year OS rates of less than 10% (25), especially for elderly patients over 60 years old, it is advisable to avoid the late neurological toxicity caused by whole-brain radiotherapy in younger patients as well. Therefore, sole WBRT is not deemed the standard initial treatment for PCNSL patients, and it should be combined with chemotherapy. In recent years, there have been numerous clinical studies investigating whether different radiotherapy regimens combined with chemotherapy could serve as first-line treatment. However, the results remain controversial (26–28). In this study, radiotherapy was recognized as a significant risk factor for PCNS-DLBCL. Patients subjected to radiotherapy experienced a 1.34 times elevated risk of mortality for OS and a 1.37 times elevated mortality for CSS relative to those without radiotherapy (*p* < 0.001). Nonetheless, multivariate analysis unveiled that radiotherapy did not stand as an independent prognostic determinant. Consequently, additional investigation is imperative to thoroughly assess the merits and drawbacks of radiotherapy.

In the past, due to the multifocal, deep-seated, and infiltrative nature of PCNSL (29), studies have shown that neither STR nor GTR provides a significant survival advantage. Instead, they are associated with higher rates of complications and mortality, along with relatively immature early tumor resection techniques (30, 31). Hence, surgical treatment has long been omitted from the recommended treatment options for PCNS-DLBCL patients and has been solely utilized for biopsy purposes to diagnose the disease. In recent years, with advancements in treatment regimens and technologies, such as the widespread utilization of MRI imaging, stereotactic techniques, and tumor visualization, have substantially enhanced the effectiveness and tolerability of surgery. Moreover, considering the lower level of evidence from early studies, traditional viewpoints have been questioned, surgical resection appears to be safe for specific cases (32, 33). After analyzing 244 publications published over 44 years, certain research suggests that there is no notable variance in complication rates between surgery and biopsy. In fact, patients undergoing GTR may even experience prolonged OS compared to those undergoing biopsy (34). Some studies also suggest that for patients younger than 70 years old with superficially located tumors, undergoing surgical resection results in significantly prolonged OS compared to those undergoing biopsy alone (median OS: 35 months vs. 8.9 months, *p* = 0.007) (15).

Our findings are consistent with this conclusion. Univariate analysis suggests that both STR and GTR are associated protective factors for survival rates in PCNSL. The HR for OS comparing STR to no surgery/biopsy is 0.77 (95% CI: 0.63–0.94), and for GTR compared to no surgery/biopsy is 0.84 (95% CI: 0.74–0.95). In terms of CSS, the HR are 0.73 (95% CI: 0.59–0.90) for STR and 0.83 (95% CI: 0.73–0.95) for GTR. Receiving GTR emerges as the exclusive independent protective factor for both OS (HR: 0.78, 95% CI: 0.69–0.89) and CSS (HR: 0.77, 95% CI: 0.68–0.88) in the multivariate regression analysis. The KM curves indicate that patients undergoing surgical resection exhibit a notable enhancement in OS or CSS, particularly among those who undergo STR. Furthermore, upon excluding patients who do not receive chemotherapy, those receiving chemotherapy in conjunction with STR demonstrate the most favorable prognosis. While multivariable Cox regression and KM analysis may appear contradictory, this discrepancy mainly stems from how they handle confounding factors. KM analysis simply compares survival probabilities over time for different groups but lacks adjustments for other influencing variables. In contrast, Cox regression can account for several variables simultaneously, adjusting for factors like age or tumor characteristics (35). Similarly, Vianda S. Stel, e.g., highlighted that discrepancies between KM analysis and Cox regression in the same dataset can be attributed to differences in how the two methods handle confounding variables (36), the study demonstrates that unadjusted KM survival curves can suggest a lower survival rate for a treatment group, while Cox regression, which adjusts for factors like age and disease stage, may show better outcomes for the same group. In our study, the unadjusted KM analysis did not take into account confounding factors such as patient age, gender, tumor location, or whether chemotherapy was administered, all of which can significantly impact survival rates. The seemingly better prognosis in the STR group may be due to more aggressive treatments (e.g., stronger chemotherapy), which compensated for the limitations of STR. Besides there may be differences in patient characteristics. Those who underwent GTR likely had less severe conditions, with tumors either smaller or located in more accessible areas, making them more suitable for complete resection. These factors were adjusted for in the Cox analysis, potentially leading to the better prognosis observed in the GTR group in the multivariate model. Thus, when interpreting survival analysis results, considering confounders is critical. Relying solely on KM curves can be misleading. In this study, Cox regression provided a more accurate reflection of the independent impact of surgical resection, indicating would like be better prognoses for GTR patients receiving chemotherapy.

This study explored the clinical characteristics of PCNS-DLBCL using a large sample and constructed a nomogram based on the SEER database and the First Affiliated Hospital of Wenzhou Medical University. The internal validation demonstrated not only good predictive accuracy but also reliable clinical applicability. Similarly, the external validation showed preferable clinical value, providing a reference for the development of clinical guidelines for PCNS-DLBCL.

There are specific constraints that apply to this research. Our study is susceptible to potential selection bias. The utilization of the SEER database led to the exclusion of certain prognostic factors, including tumor size, Karnofsky Performance Status score, and unknown or missing serum lactate dehydrogenase levels. Finally, detailed information on chemotherapy or radiotherapy regimens was not provided, and the extent of surgical resection (STR and GTR) was not quantified.

## Conclusion

In summary, age, gender, marital status, tumor location, chemotherapy, HIV infection status, and surgical intervention are separate prognostic factors for OS in PCNS-DLBCL patients. Gender does not emerge as an independent prognostic factor for CSS, whereas radiotherapy solely impacts OS and CSS and is not an independent prognostic factor for PCNS-DLBCL patients. Surgical resection may confer survival benefits for PCNS-DLBCL patients, especially when combined with chemotherapy and different extents of resection, improving both OS and CSS. Hence, the importance of surgery in treating PCNS-DLBCL should be reevaluated, and personalized surgical strategies should be developed according to the unique characteristics of each patient.

## Data Availability

The datasets presented in this study can be found in online repositories. The names of the repository/repositories and accession number(s) can be found in the article/Supplementary material.
